# A case of recurrent leg necrotic ulcers secondary to silicone migration in a transgender patient: radiographic, ultrasound and MRI findings

**DOI:** 10.1259/bjrcr.20150309

**Published:** 2016-10-28

**Authors:** Eugen Lungu, Ariane Thibault-Lemyre, J Manuel Dominguez, Dominique Trudel, Nathalie J Bureau

**Affiliations:** ^1^ Faculty of Medicine, Université de Montréal, Montréal, Canada; ^2^ Department of Medicine, Université de Montréal, Montréal, Canada; ^3^ Department of Pathology, Centre Hospitalier de l´Université de Montréal, Montréal, Canada; ^4^ Radiology Department, Centre Hospitalier de l´Université de Montréal, Montréal, Canada

## Abstract

Injection of massive quantities of liquid silicone into body parts for cosmetic purposes is a popular practice among the transgender population. Although a myriad of short-term complications associated with this procedure have been described, few reports of chronic and persistent ailments exist. We present the case of a male-to-female transgender with recurrent necrotic leg ulcers associated with migration of the silicone material injected in the buttocks 25 years ago. We review the imaging findings as well as the clinical and pathological aspects of this presentation, with an emphasis on the necessity of a high degree of suspicion for silicone-associated complications in a transgender patient presenting with leg wounds. We highlight the importance of the characteristic sonographic *snowstorm* artefact generated by free silicone material in soft tissues in the diagnosis of this entity.

## Summary

Liquid silicone injections have been employed as a soft-tissue contour augmentation procedure for cosmetic purposes for the past five decades.^1^ Although recognized as a safe and effective method of achieving corrective results when employed in a standardized manner by skilled professionals, it can have catastrophic consequences when unlicensed individuals make use of materials of subpar quality.^1^ Several complications associated with illegal injection of silicone have been extensively reported and focus mostly on acute events, such as local reactions including cellulitis, ulcerations and nodule formation, as well as migration of the injected material, infection and death from cardiorespiratory collapse associated with pneumonitis and alveolar haemorrhage.^1^^–^^8^ Illicit services continue to appear enticing to individuals seeking low-budget enhancement of body parts, and patients are often reluctant to disclose a history of such procedures.^8^ We present the case of a patient suffering from chronic necrotic lower leg ulcers 25 years following the injection of silicone in the buttocks. Although leg ulcerations associated with local silicone injections have been previously reported,^9^^–^^11^ to our knowledge, no cases of persistent, recurrent leg ulcerations following migration of the injected material in the gluteal area after such an extensive period have been described. Knowledge of the imaging manifestations of this entity should warrant consideration for silicone-associated complications in the differential diagnosis of lower-extremity ulcers, especially in a transgender patient.

## Case presentation

Written informed consent was obtained from the patient for publication of this case report, including accompanying images. A 43-year-old transgender male-to-female sex worker with a history of hepatitis C infection and smoking presented to our tertiary university health centre complaining of a right lower extremity painful lesion. Onset of the pain occurred 1 month ago, along with progressive development of necrotic skin changes without constitutional symptoms. The patient reported a history of bilateral gluteal and mammary injections with unidentified liquid silicone-like material 25 years ago by a non-medical individual in North Africa. She described several similar episodes of soft-tissue ulcers in her legs following minor trauma such as mosquito bites occurring over the years. According to the patient, the previous acute episodes resolved following antibiotic treatment without surgical debridement but subsequently led to the development of persistent scarred ulcers.

**Figure 1.**
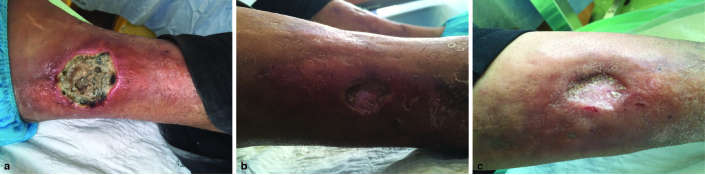
(a) Photograph of the medial aspect of the right leg of a 43-year-old male-to-female transgender with migration of silicone material to the legs following silicone injections in the buttocks showing a cutaneous necrotic ulcer following partial debridement. (b) Lateral view of the right and the left leg (c) showing evidence of scarred cutaneous ulcers.

Inspection of the right leg revealed a 3.5 × 3.0 × 0.8 cm necrotic skin ulcer proximal to the medial malleolus (Figure 1) with foul-smelling serous discharge and diffuse erythema of the foot. Several scarred skin ulcers were also noted along both legs. Diffuse cutaneous induration was present. Palpation of the lower extremity pulses was equivocal.

## Imaging

**Figure 2.**
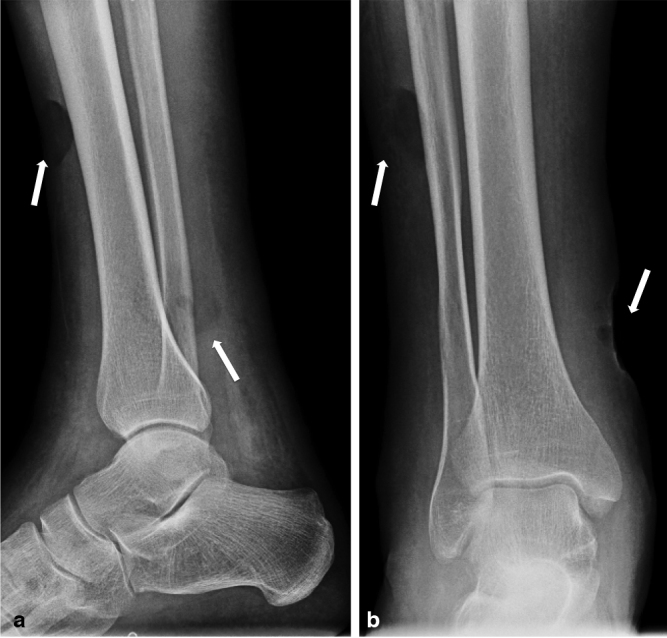
(a) Lateral and (b) frontal radiographs of the right leg demonstrating diffuse thickening and reticular, micronodular infiltration of the subcutaneous soft tissues and the presence of two cutaneous ulcerations (arrows) without evidence of soft-tissue calcification or osteomyelitis.

**Figure 3.**
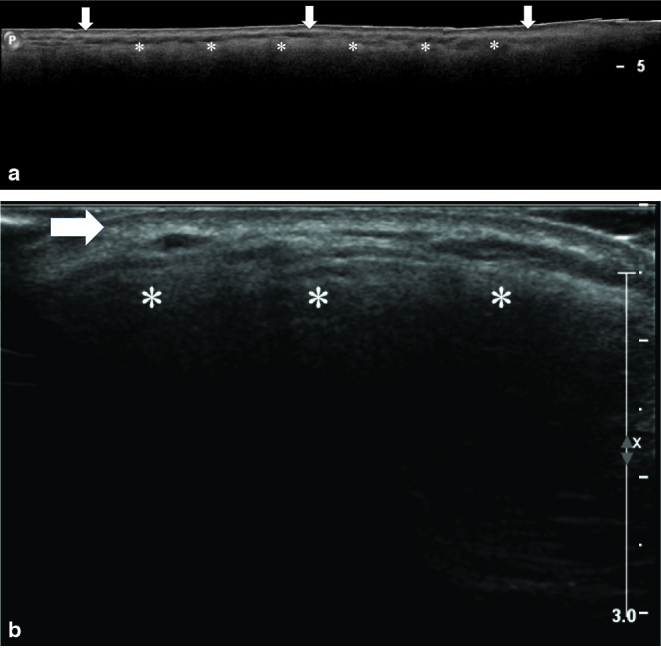
(a) Longitudinal extended-field-of-view ultrasono-graphy scan of the anterolateral aspect of the right leg and (b) transverse ultrasonography scan of the anterolateral aspect of the left leg showing normal appearance of the dermis (arrows) and diffuse hyperechogenicity of the subcutaneous tissues (asterisks) with strong posterior acoustic shadowing masking the deeper structures. This “snowstorm pattern” is consistent with liquid silicone filler.

**Figure 4.**
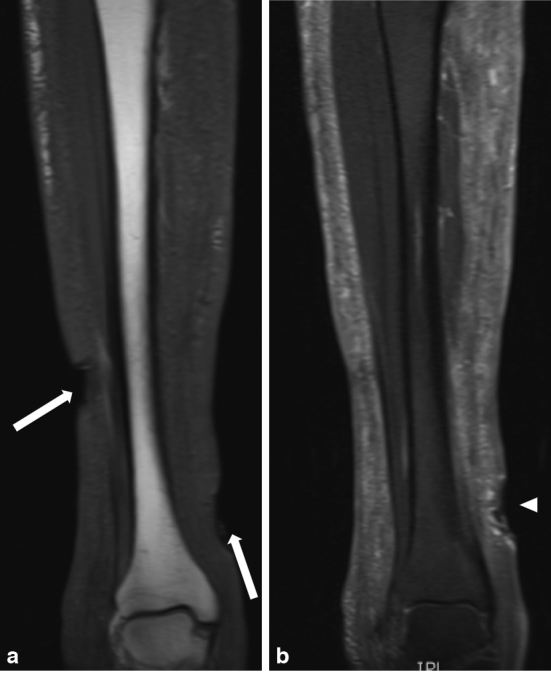
(a) Coronal *T*_1_ weighted image of the right leg depicting diffuse intermediate signal of the subcutaneous tissues and two cutaneous ulcers (arrows). (b) Slightly more anterior coronal short tau inversion-recovery image showing diffuse micronodular hyperintense signal in the cutaneous and subcutaneous tissues and the skin ulcer proximal to the medial malleolus (arrowhead). The osseous structures and muscles are intact without evidence of infection. There is no ferromagnetic artefact or other evidence of foreign body material.

Radiography (Figure 2), ultrasonography (Figure 3) and MRI (Figure 4) of the right leg were performed to rule out osteomyelitis and assess the extent of soft tissue involvement. There was no radiographical evidence of osteomyelitis or foreign body material. On ultrasonography, extensive homogeneous acoustic shadowing, characteristic of the *snowstorm* artefact, obscured the deep subcutaneous tissues and muscle compartments of both legs, thus precluding diagnostic evaluation. The MRI demonstrated diffuse *T*_1_ intermediate signal and short tau inversion-recovery (STIR) increased signal of the cutaneous and subcutaneous tissues but did not show any evidence of foreign body material or osteomyelitis.

In anticipation of surgical debridement of the ulcer, a lower extremity duplex arterial and venous ultrasonography was performed in light of the equivocal peripheral pulses and long-term use of hormonal therapy. Diffuse acoustic shadowing impeded an optimal evaluation; only the popliteal artery was visualized and it exhibited a biphasic waveform pattern, suggesting acceptable blood flow.

## Laboratory results

The patient did not present any clinical or biochemical evidence of hypercalcaemia that would have suggested a foreign body granulomatous reaction. Culture of the ulcer showed infection by oxacillin-sensitive *Staphylococcus aureus*.

## Treatment

The patient received a combined 14-day course of intravenous and oral antibiotherapy with a first-generation cephalosporin. In spite of the limited assessment of the vascularity of the right lower extremity, surgical debridement was undertaken. Wound care consisting of washing with normal saline solution and application of hydrocolloid, foam and chlorhexidine dressings was subsequently performed.

## Follow-up and histopathological manifestations

**Figure 5.**
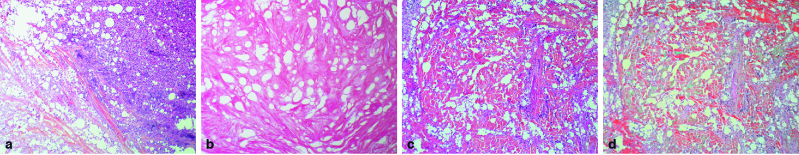
Haematoxylin, phloxine and saffron stained-images of specimen sampled from the ulcer displaying tissue abscess formation (a) and devitalization (b). Numerous micro- and macrovacuoles (c) without refractile or refringent material (d) are consistent with amorphous material deposition.

Approximately 1 month following discharge, the patient returned because of aggravation of the leg ulcer and increasing pain, and subsequently underwent more extensive debridement. A histological specimen (Figure 5) was sampled perioperatively and demonstrated extensive tissue devitalization with abscess formation and epidermal ulceration. Noticeably, the dermis contained numerous micro- and macrovacuoles, without evidence of refractile or refringent material. No foreign body reaction or boxcar-like bacteria were identified.

## Discussion

Injectable silicone has been employed for soft-tissue augmentation in North America since the 1960s, and has been hailed for its inertness, ease of use and low cost.^1,8^ Migration of improperly injected or poor quality silicone through tissue planes has been associated with numerous complications, ranging from formation of benign cutaneous nodules to death.

Lower extremity complications occur primarily at the injection sites at the level of the gluteal and calf areas. Rae et al^11^ presented the case of a female exhibiting chronic bilateral leg ulcerations 23 years following the injection of silicone into her calves. Agrawal et al^9^ reported a case of granuloma formation developing at the site of a silicone injection into the buttocks leading to hypercalcaemia. Lee and Choi^10^ described the development of multiple *in situ* calcifications and skin defects 30 years after silicone injection into the lower legs.

Our patient did not present any clinical abnormality at the location of the initial silicone injections in the peritrochanteric and gluteal regions, but developed multiple, recurrent infected and necrotic skin ulcerations in both legs, a finding consistent with gravitation of the injected material into more dependent areas. Migration of the silicone material is thought to occur through tissue planes when large quantities are injected at once.^4^

Radiographic features of silicone injection in the buttocks have been described as abnormal soft-tissue densities caused by radiodense injected material or secondary dystrophic calcifications.^12^ Our case demonstrated diffuse thickening of the subcutaneous tissues with a dense, non-specific, infiltrative, micronodular pattern consistent with disseminated silicone droplets.

On MRI, the appearance of silicone injection has been reported as low signal on *T*_1_ and high signal on *T*_2_ weighted images associated with either a nodular or diffuse pattern.^12,13^ Some authors have advocated the use of STIR sequences with water suppression to increase the contrast between water, fat and silicone or STIR sequences with spectral suppression of silicone to confirm the material as silicone and distinguish it from other high signal *T*_2_ lesions such as cysts or abscesses.^13^ Reflecting the presence of silicone droplets that had migrated from a remote injection site, the present case showed a diffuse micronodular and reticular pattern in the subcutaneous tissues, intermediate signal on *T*_1_ and high signal on STIR images.

The distinctive ultrasonography *snowstorm *artefact of liquid silicone dispersed in the soft tissues following extracapsular rupture of breast implants has long been recognized,^14^ and more recently, has been studied experimentally.^15^ In the present case, this characteristic artefact was most likely caused by the free liquid silicone droplets dispersed in the soft tissues that generated highly reflective interface with acoustic shadowing.

Histological evaluation of soft tissues infiltrated by silicone can show numerous vacuoles containing refractile, non-birefringent material.^11,16^ In our case, no refractile material was noted within the vacuoles, suggesting that solvents used in the preparation of the specimen may have degraded the silicone. The histopathological findings of the present case discord with the growing body of evidence incriminating a host inflammatory-type response for the damage inflicted on the soft tissue.^1,8,9,17^ Rather, it is possible that haematogenous seeding of injected material from distant bacterial infections may lead to local changes.^8^ It is also plausible that minor trauma to the lower extremity, for example, mosquito bites, as reported by the patient, could facilitate and perpetuate an infectious process.

Treatment strategies of necrotic lower extremity wounds secondary to silicone migration and options to prevent recurrences are limited owing to lack of evidence-based recommendations. Surgical excision of the infiltrated areas has been previously attempted, mainly with unsatisfactory results owing to the inability to completely remove the injected material.^18,19^ The widespread dissipation of silicone puts the patient at great risk of additional ulcer recurrences.

Although we did not identify any formal data regarding the prevalence of complications related to silicone injections in the gluteal region or other body areas in transgender individuals, several studies are dedicated to underlining the devastating outcome of such practices in this population.^2,9,17,19^ This case illustrates the necessity of a high index of clinical suspicion when evaluating ulcers in a transgender patient. Because radiographical and MRI findings are generally non-specific, unless potentially employing silicone-specific MRI sequences if the diagnosis is known or suspected, ultrasonography evaluation proves invaluable.

## Learning points

1. Injection of large amounts of silicone for cosmetic purposes is associated with multiple short- and long-term complications at either the local or remote anatomical sites and is more prevalent in the transgender community.
2. When evaluating ulcers in a transgender patient, silicone injection-associated complications should be considered as a possible aetiology.
3. Radiographical features of silicone injections are non-specific densities or diffuse thickening of the subcutaneous tissues.
4. Dispersal of free silicone droplets in the soft tissue casts a characteristic *snowstorm *acoustic shadowing artefact on ultrasonography.
5. Injected silicone material displays a non-specific micronodular or reticular pattern of low-to-intermediate signal intensity on *T*_1 _and high signal intensity on *T*_2_ weighted images.
